# Inhibition of histone deacetylase 2 reduces MDM2 expression and reduces tumor growth in dedifferentiated liposarcoma

**DOI:** 10.18632/oncotarget.27144

**Published:** 2019-10-01

**Authors:** Nathan D. Seligson, Colin W. Stets, Bryce W. Demoret, Achal Awasthi, Nicholas Grosenbacher, Reena Shakya, John L. Hays, James L. Chen

**Affiliations:** ^1^ Department of Pharmacy, The Ohio State University Wexner Medical Center and Comprehensive Cancer Center, Columbus, Ohio, USA; ^2^ Department of Biomedical Informatics, The Ohio State University, Columbus, Ohio, USA; ^3^ Target Validation Shared Resource, The Ohio State University Wexner Medical Center and Comprehensive Cancer Center, Columbus, Ohio, USA; ^4^ Division of Medical Oncology, Department of Internal Medicine, The Ohio State University, Columbus, Ohio, USA; ^5^ Division of Gynecologic Oncology, Department of Obstetrics and Gynecology, The Ohio State University, Columbus, Ohio, USA

**Keywords:** dedifferentiated liposarcomas, MDM2, HDAC2, romidepsin, MI-192

## Abstract

Dedifferentiated liposarcoma (DDLPS) is a highly morbid mesenchymal tumor characterized and driven by genomic amplification of the *MDM2* gene. Direct inhibition of MDM2 has shown promise pre-clinically, but has yet to be validated in clinical trials. Early *in vitro* studies have demonstrated that pan-histone deacetylase (HDAC) inhibition may have anti-MDM2 effects. Here we present *in silico*, *in vitro*, and mouse xenograft studies that suggest that specifically targeting HDAC2 reduces MDM2 expression and has anti-tumor affects in DDLPS. Two independent datasets, The Cancer Genome Atlas (TCGA; *n* = 58) and the Memorial Sloan-Kettering Cancer Center Dataset (MSKCC; *n* = 63), were used to identify the co-expression between class I *HDACs* and *MDM2*, and their clinical impact. *HDAC*2 was highly co-expressed with *MDM2* (TCGA: Spearman’s coefficient = 0.29, *p* = 0.03; MSKCC: Spearman’s coefficient = 0.57, *p* < 0.001). As both a continuous and dichotomous predictor, elevated *HDAC2* expression was associated with worsened disease-free survival in the TCGA (Continuous: Hazard-ratio (HR) 1.7; 95% Confidence Interval (95%CI) 0.97–2.9; *p* = 0.06; Dichotomous: HR 7.1, 95%CI 2.5–19.8, *p* < 0.001) and distant recurrence-free survival in the MSKCC (Continuous: HR 2.2; 95%CI 1.1–4.8; *p* = 0.04; Dichotomous: HR 2.8, 95%CI 1.2–6.4, *p* = 0.02). *In vitro*, treatment of DDLPS cell lines with the HDAC inhibitors MI-192 (HDAC2/3 inhibitor) or romidepsin (HDAC1/2 inhibitor) reduced *MDM2* expression and induced apoptosis. In a murine DDLPS xenograft model, romidepsin reduced tumor growth and lowered tumor MDM2 expression. RNA-sequencing of romidepsin treated mouse tumors demonstrated markers of TP53 reactivation. Taken together, our data supports the hypothesis that targeting HDAC2 may represent a potential strategy to modulate MDM2 expression in DDLPS.

## INTRODUCTION

Dedifferentiated liposarcoma (DDLPS) is a highly morbid mesenchymal tumor accounting for approximately 20% of all soft-tissue sarcomas [1]. A hallmark of DDLPS is the genomic amplification of *Mouse Double Minute 2 Homolog* (*MDM2*) [2–5]. MDM2 degrades p53, thus, an amplification in *MDM2* results in reduced p53 activity and a shift towards pro-survival pathways. Our previous work has demonstrated that the amplification of *MDM2* is directly tied to biological activity and clinical response to chemotherapy in this disease [6, 7]; furthermore, eliminating or reducing MDM2 activity may reduce of the oncogenicity of DDLPS tumors [7, 8]. A primary strategy to target MDM2 in DDLPS has been to sterically inhibit the ability of MDM2 to bind p53 [8–11]. These treatment modalities have shown promise pre-clinically but have yet to be proven clinically viable.

Recent *in vitro* studies suggest that pan-histone deacetylase (HDAC) inhibitors may modestly reduce MDM2 expression [12, 13] but have not been fully evaluated in DDLPS. While targeted HDAC inhibitors have been approved in the treatment of hematologic malignancies, they have shown limited activity in a number of solid tumor types with notable toxicities [14–18]. A benefit of targeted HDAC inhibition clinically would be the potential to avoid off-target toxicities associated with pan-HDAC inhibition. Whether targeted HDAC inhibition with defined specificity would be efficacious in DDLPS has yet to be explored.

The HDAC family of proteins is vast and is comprised of four groups and 18 members. To this end, our *in silico* analysis identified HDAC2 as a potential target for modulation of MDM2 in DDLPS. A recent study assessing gene expression in 1,332 mesenchymal tumors and normal tissues identified a specific increase in *HDAC2* expression in DDLPS compared to other soft tissue sarcomas (STS) and normal tissue [19]. In lung cancer cell lines, MDM2 expression could be reduced by selectively knocking down *HDAC2* [20]. In this study we demonstrate that selective inhibition of HDAC2 induced p53-dependent survivin downregulation through MDM2 proteasomal degradation [20]. Results presented here suggest that inhibition of HDAC2, specifically utilizing the HDAC1/2 inhibitor romidepsin, reduces MDM2 expression and promotes apoptosis in DDLPS. This may serve as a viable therapeutic modality for this highly morbid disease.

## RESULTS

### HDAC2 is positively correlated with MDM2 expression in DDLPS samples

To determine the co-expression of HDAC family members and *MDM2*, we evaluated mRNA expression data from two independent DDLPS datasets for *MDM2* and members of the class I HDAC family of genes [21]. In both The Cancer Genome Atlas (TCGA) and the Memorial Sloan-Kettering Cancer Center (MSKCC) datasets, *HDAC2* was most positively correlated of the HDAC family members to *MDM2* (TCGA: Spearman’s coefficient = 0.29, *p* = 0.03; MSKCC: Spearman’s coefficient = 0.57, *p <* 0.001; Table 1; Supplementary Figure 1).

**Table 1:** Class I Histone Deacetylate (HDAC) mRNA co-expression with *MDM2*

Class I HDACs	Cytoband	The Cancer Genome Atlas (TGCA)		Memorial Sloan-Ketting Cancer Center Dataset (MSKCC)	
		Spearman’s correlation^†^	*p*-value	Spearman’s correlation^†^	*p*-value
*HDAC1*	1p35.2-p35.1	-	>0.1	-	>0.1
*HDAC2*	6q21	0.29	0.03	0.57	<0.001
*HDAC3*	5q31.3	0.23	0.09	-	>0.1
*HDAC8*	Xq13.1	-	>0.1	-	>0.1

^†^Correlations with a significance level of >0.1 not shown.

### Elevated HDAC2 levels are poorly prognostic in DDLPS

**Figure 1:**
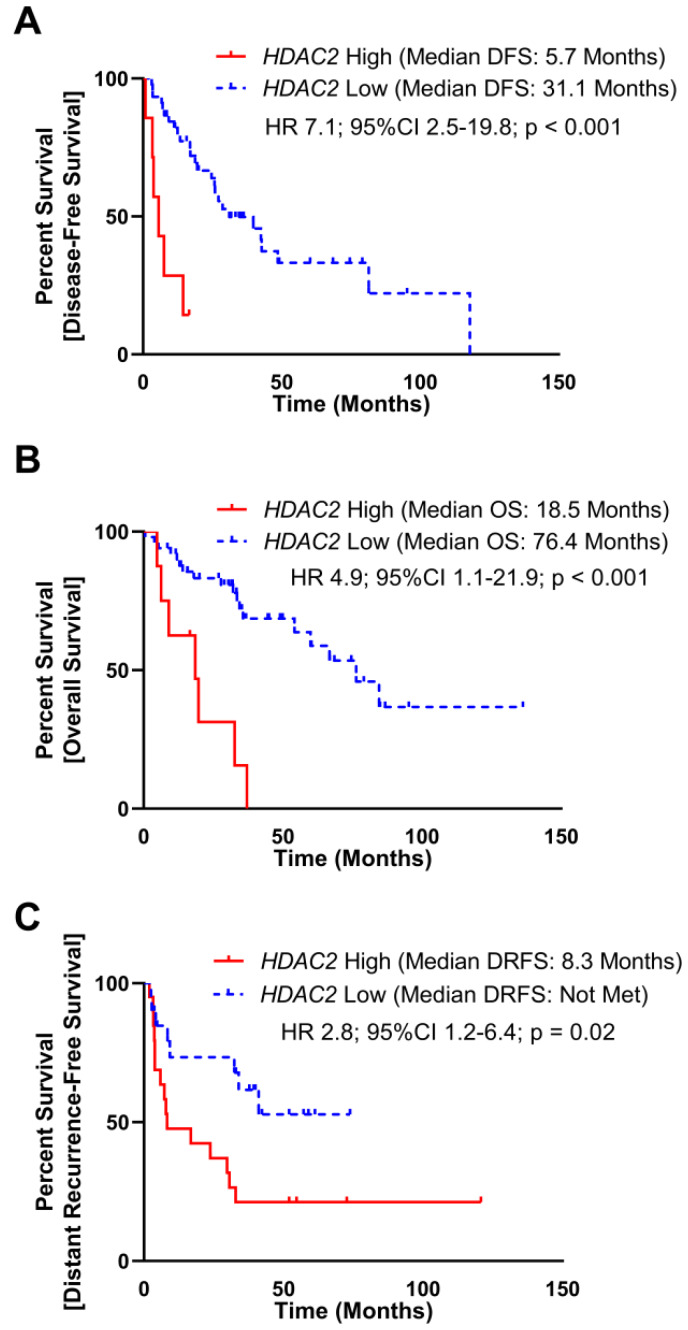
Elevated *HDAC2* mRNA expression is poorly prognostic in DDLPS. (**A**) mRNA expression of *HDAC2* and disease-free survival (DFS) data from 52 subjects with DDLPS from The Cancer Genome Atlas (TCGA) were split into two groups utilizing maximally selected rank statistic (Supplementary Figure 1A). Subjects with elevated *HDAC2* expression experienced reduced DFS (median DFS: *HDAC2* High 5.7 months, *HDAC2* Low 31.1 months; HR 7.1, 95%CI 2.5–19.8, *p* < 0.001). (**B**) mRNA expression of *HDAC2* and overall survival (OS) data from 58 subjects with DDLPS from the TCGA were split into two groups utilizing maximally selected rank statistics (Supplementary Figure 1A). Subjects with elevated *HDCA2* expression experienced reduced OS (median OS: *HDAC2* High 18.5 months, *HDAC2* Low 76.4 months; HR 4.4, 95%CI 1.1–21.9, *p* = 0.04). (**C**) mRNA expression of *HDAC2* and distant recurrence-free survival (DRFS) data from 40 subjects with DDLPS from the Memorial Sloan-Kettering Cancer Center Dataset (MSKCC) were split into two utilizing maximally selected rank statistic (Supplementary Figure 1B). Subjects with elevated *HDAC2* expression experienced reduced DRFS (median DRFS: *HDAC2* High 8.3 months, *HDAC2* Low not met; HR 2.8, 95%CI 1.2–6.4, *p* = 0.02).

To assess the clinical significance of *HDAC2* expression, we evaluated TCGA and MSKCC datasets, which included mRNA expression and DFS. As a continuous variable in the TCGA dataset, elevated *HDAC2* expression was associated with a reduced disease-free survival (DFS) manifested as an increase in hazard of 5% per increment of *HDAC2* reads (Hazard-ratio (HR) 1.7; 95% Confidence Interval (95%CI) 0.97–2.9; *p* = 0.06). To assess *HDAC2* expression as a dichotomous predictor of DFS, subjects were stratified into *HDAC2* High and *HDAC2* Low groups utilizing a maximal inflection point test to identify the optimal cut off using both gene expression and DFS time to separate cohorts (methods, Supplementary Figure 2A), elevated *HDAC2* was associated with poor DFS (median DFS: *HDAC2* High 5.7 months, *HDAC2* Low 31.1 months; HR 7.1, 95%CI 2.5–19.8, *p <* 0.001; Figure 1A). Elevated *HDAC2* expression also correlated with poor overall survival (OS) as a continuous (HR 1.06; 95%CI 1.01–1.11; *p* = 0.02) and dichotomous predictor (median OS: *HDAC2* High 18.5 months, *HDAC2* Low 76.4 months; HR 4.9, 95%CI 1.1–21.9, *p <* 0.001; Figure 1B).

In the MSKCC dataset, elevated *HDAC2* expression as a continuous variable was associated with a reduced distant recurrence-free survival (HR 2.2; 95%CI 1.1–4.8; *p* = 0.04). As a dichotomous predictor stratified at the maximal inflection point (Supplementary Figure 2B), elevated *HDAC2* was associated with poor DRFS (median DRFS: *HDAC2* High 8.3 months, *HDAC2* Low not met; HR 2.8, 95%CI 1.2–6.4, *p* = 0.02; Figure 1C). The MSKCC dataset did not include OS data.

### HDAC2 knockdown reduces MDM2 expression in DDLPS

To interrogate the effect of modulating *HDAC2* on the expression of MDM2 we used *shRNA* targeted to *HDAC2* in the DDLPS cell line LPS246. The addition of *shRNA* reduced HDAC2 protein expression compared to scrambled *shRNA* control. MDM2 expression was reduced in *shRNA* treated cells compared to scrambled *shRNA* control (Supplementary Figure 3).

### Pharmacologic inhibition of HDAC2 reduces cell proliferation

**Figure 2:**
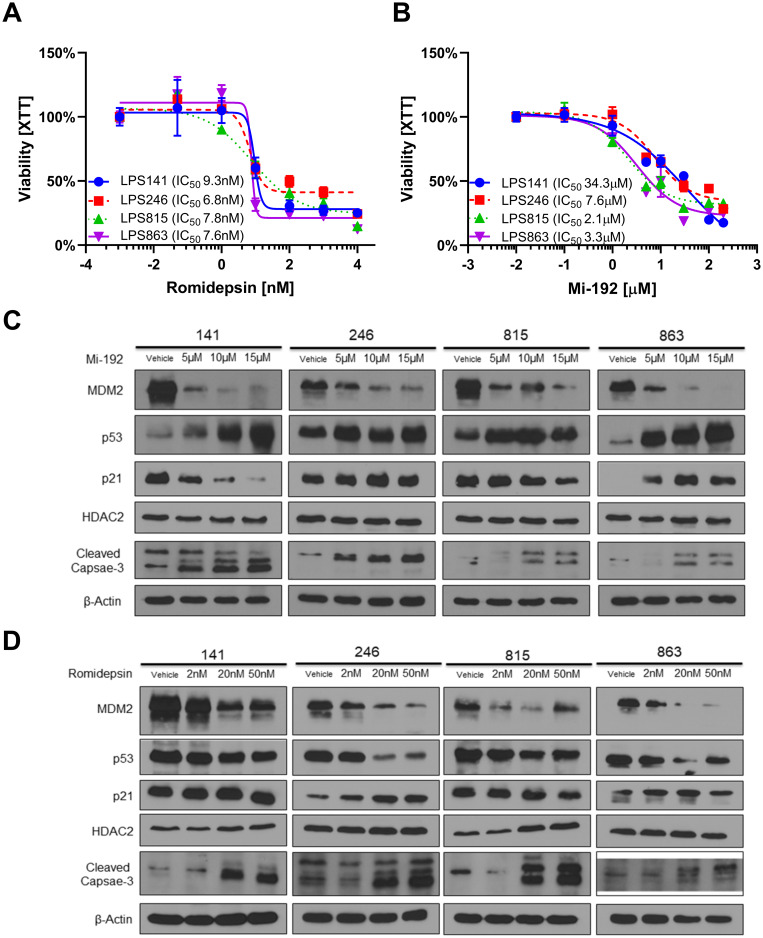
HDAC2 inhibitors reduce MDM2 expression in *in vitro* models of DDLPS. (**A**, **B**) Cellular viability in a panel of four DDLPS cell lines as measured by the XTT assay. Percent viability is relative to vehicle treated control. Cellular sensitivity to romidepsin (A) and MI-192 (B) did not correlate with baseline MDM2 status in these cells. (**C**) Protein expression for DDLPS cells treated for 48 hours with MI-192. MI-192 reduced MDM2 and p21 expression while increasing expression of p53 and cleaved capsase-3. (**D**) Protein expression for DDLPS cells treated for 48 hours with romidepsin. Romidepsin reduced MDM2 expression while increasing expression cleaved capsase-3. In the 246 and 863 cell lines, romidepsin reduced p53 expression.

To assess the effect of more targeted HDAC inhibition in DDLPS, we used a panel of four DDLPS cell lines whose MDM2 status was fully outlined previously [8]. Cells were treated with two HDAC inhibitors, either MI-192 (specific to HDAC2 and HDAC3) or romidepsin (specific to HDAC1 and HDAC2), for 72 hours [22, 23]. All cell lines treated with MI-192 or romidepsin showed similar sensitivity with IC_50_’s in the low micromolar (IC_50_ range: 2–34 μM) and nanomolar (IC_50_ range: 6–9 nM) range respectively and did not correlate with baseline MDM2 status (Figure 2A–2B).

### HDAC inhibitors reduced MDM2 protein *in vitro* and activated p53-mediated cell death

To assess the molecular effect of HDAC2 inhibition in DDLPS, DDLPS cell lines were treated with either MI-192 or romidepsin at increasing doses for 48 hours before protein was collected to be measured by Western blot. Both MI-192 and romidepsin significantly reduced MDM2 protein expression compared to vehicle control with little effect on HDAC2 protein expression (Figure 2C–2D). MI-192 induced an increase in p53 expression; however, this effect was not seen consistently in romidepsin treated DDLPS cell line models. Finally, both MI-192 and romidepsin increased expression of cleaved-caspase 3, suggesting an induction of cellular apoptosis.

### HDAC2 inhibition elicits anti-tumor effect and decreases MDM2 RNA levels in DDLPS xenografts

**Figure 3:**
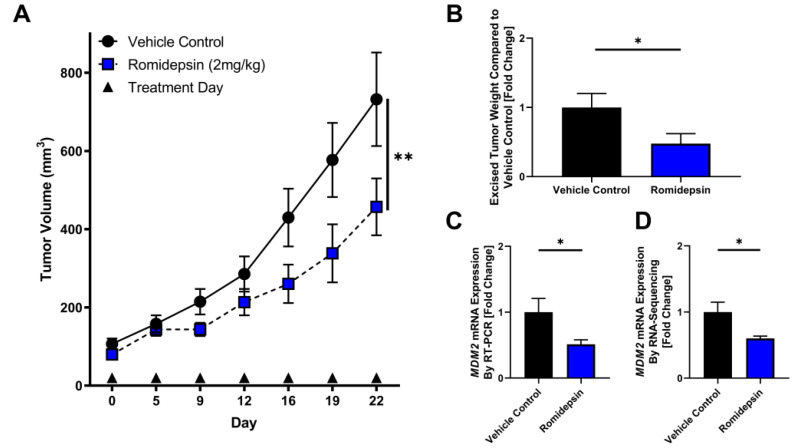
Romidepsin exhibits anti-tumor effect in xenograft model of DDLPS. (**A**–**C**) DDLPS xenograft models with bilateral flank injections of the LPS863 cell line were randomly divided into treatment with vehicle control or romidepsin. (A) Tumor growth was significantly reduced in mice treated with romidepsin compared to vehicle control (Day 22: control 357.7 ± 231.7 mm^3^, romidepsin 233.8 ± 130.3 mm^3^; *p* = 0.001). (**B**–**D**) Mice were sacrificed at day 22 and tumors excised for further analysis. (B) Excised tumors were weighed, demonstrating a lower mean tumor weight in mice treated with romidepsin compared to control (fold change compared to control: control 1.0 ± 0.2, romidepsin 0.48 ± 0.14; *p* = 0.04). (C) mRNA was collected from excised tumors and measured for *MDM2* expression normalized to B2M. Romidepsin significantly lowered *MDM2* expression compared to vehicle control (fold change compared to control: control 1.0 ± 0.21, romidepsin 0.51 ± 0.07; *p* = 0.02). (D) mRNA was collected from excised tumors and measured for *MDM2* expression by RNA-sequencing. Romidepsin significantly lowered *MDM2* expression compared to vehicle control (fold change compared to control: control 1.0 ± 0.15, romidepsin 0.60 ± 0.03; *p* = 0.04). ^*^ *p* < 0.05; ^**^ *p* < 0.01.

To test the effect of HDAC2 inhibition *in vivo*, we utilized a DDLPS mouse model subcutaneously xenografted with the LPS863 cell line. When tumors were palpable, mice were randomized to receive either vehicle control or romidepsin injected IP twice weekly. In DDLPS xenografts, romidepsin significantly delayed tumor growth as a single agent compared to vehicle control (Day 22: control 357.7 ± 231.7 mm^3^, romidepsin 233.8 ± 130.3 mm^3^; *p* = 0.001; Figure 3A). In excised tumors, romidepsin reduced tumor weight (fold change compared to control: control 1.0 ± 0.2, romidepsin 0.48 ± 0.14; *p* = 0.04; Figure 3B) and reduced *MDM2* levels as measured by quantitative RT-PCR (fold change compared to control: control 1.0 ± 0.21, romidepsin 0.51 ± 0.07; *p* = 0.02; Figure 3C). No appreciable mouse toxicity in terms of body weight or fur changes were noted.

Four tumors from both vehicle control and romidepsin treated mice were selected at the time of tumor harvest for further analysis utilizing RNA sequencing. *MDM2* was significantly reduced in tumors in the romidepsin arm compared to vehicle control (fold change compared to control: control 1.0 ± 0.15, romidepsin 0.60 ± 0.03; *p* = 0.04). To assess global regulatory changes induced by romidepsin treatment, Ingenuity Pathway Analysis was used to assess the most highly altered regulatory pathways by treatment group. Top altered regulators included that of the TP53 network (Listing of altered regulators, Supplementary Table 1).

## DISCUSSION

Advanced DDLPS is associated with both poor prognosis and poor response to treatment. Pathognomonic to all DDLPS is the amplification of the *MDM2* gene whose direct effect is to inhibit p53 activity [6, 24]. Pre-clinical studies have demonstrated that *MDM2* action may be targetable; however, clinical development remains an on-going challenge with notable toxicity [8–11]. Here we demonstrate an alternate approach through the inhibition of HDAC2. This HDAC2 inhibition reduces MDM2 expression and abrogates tumor growth *in vitro* and *in vivo*.

We established HDAC2 as an epigenetic protein of interest in two independent datasets using baseline mRNA expression of DDLPS tumors. *HDAC2* was the most highly co-expressed class I HDAC with *MDM2*. In these datasets, elevated *HDAC2* was associated with poor DFS, DRFS, and OS. Clearly prospective studies will be necessary to identify the clinical and prognostic utility of *HDAC2* expression in DDLPS. Our data does suggest that HDAC2 may play a role in the biology of DDLPS. We acknowledge that results from this study cannot be used to determine an appropriate *HDAC2* mRNA clinical cutoff due to a lack of a reproducible sequencing platform between datasets and the need of a larger database of DDLPS patients from which to draw.

A prior study of HDAC inhibition demonstrated *in vitro* effect of pan-HDAC inhibition in DDLPS [12]. Our work has further evaluated HDAC2 specific medications MI-192 and romidepsin. Excitingly, romidepsin is an FDA approved agent in the treatment of other malignancies. *In vitro*, romidepsin showed strong effect in our panel of DDLPS cell lines, effectively reducing MDM2 expression while triggering apoptosis. *In vivo*, romidepsin demonstrated significant activity in a xenograft mouse model of DDLPS. Importantly, romidepsin treated tumors not only demonstrated significantly smaller tumors but also exhibited a reduction in MDM2 expression compared to vehicle control. Upstream regulatory analysis identified reactivation of p53 activity that may be a critical driver of romidepsin’s anti-tumor effects in DDLPS. Taken together, this pre-clinical analysis suggests that inhibition of HDAC2, specifically utilizing the HDAC1/2 inhibitor romidepsin, reduces MDM2 expression and may promote apoptosis in DDLPS. We have previously shown that *MDM2* amplification and expression is associated with chemoresistance in DDLPS [7]. Further study of the HDAC2:MDM2 axis may provide further insight into mechanisms of chemoresistance in this disease. Additionally, HDAC2 modulation of MDM2 along with other DNA damaging agents and CDK4 inhibitors are under development.

In summary, to our knowledge, this is the first report of romidepsin's activity in MDM2-amplified tumors [16]. The data presented here suggests a potential role for HDAC2 inhibition in DDLPS as a modulator of the MDM2:p53 pathway. Further clinical trials will be needed to verify this hypothesis.

## MATERIALS AND METHODS

### Clinical datasets

*The Cancer Genome Atlas (TCGA)* - Gene expression, as measured by RNA-seq, from 58 subjects with DDLPS from the TCGA and was collected and assessed utilizing cbioportal [25]. Disease-free survival (DFS) data was available for 52 subjects, while overall survival (OS) data was available for all 58 subjects. *HDAC2* was considered as both a continuous and dichotomous predictor of clinical outcomes.

*Memorial Sloan-Kettering Cancer Center Dataset (MSKCC)* - Gene expression data from 40 subjects with DDLPS was downloaded from the GEO database as previously described [26]. Gene expression was measured by microarray. Distant recurrence-free survival (DRFS) and gene expression was collected for all 40 subjects. *HDAC2* was considered as both a continuous and dichotomous predictor of clinical outcomes.

### Cell culture

Patient-derived DDLPS cell lines (LPS246, LPS815, and LPS863) were brought directly into culture from patients from the SARC Sarcoma SPORE housed at The Ohio State University and maintained as previously described [8]. Dr. Jonathan Fletcher (Brigham and Women’s Hospital, Boston, MA, USA) generously provided us with LPS141 cells.

### Chemical compounds and reagents

Romidepsin (Selleckchem) and MI-192 (Sigma) were dissolved in DMSO as recommended by the manufactures and stored at −80° C for *in vitro* experiments. Serial dilutions were made to ensure a final DMSO concentration was below 0.1%.

### Proliferation assay

Exponentially growing DDLPS cell lines were seeded into 96-well plates and treated with the indicated compounds. Cell viability was determined by adding 20 μL of XTT reagent (Roche) as per manufacturer’s instructions. Romidepsin treatments ranged from 0.001–10 μM while MI-192 treatments ranged from 1–100 μM.

### Western blotting

In 100 mm culture dishes, 750,000 cells were plated and allowed to attach overnight. Drug (MI-192, Romidepsin) concentrations were calculated based on the IC_50_ concentration of the DDLPS cell lines to each respective drug. Cells were lysed using SDS/laemmli lysis buffer. Lysates were clarified by sonication. Supernatant was used for protein analysis. Equal amount (25 ug) of cell extracts were resolved by SDS-polyacrylamide gel electrophoresis and transferred to a pretreated PVDF membrane for 90 minutes in a cold room. Membranes were incubated overnight at 4° C using Rabbit monoclonal antibodies of MDM2 (ThermoFisher), HDAC2 (Abcam), P53 (Santa Cruz), P21 (Santa Cruz), and Caspase 3 (Thermofisher), using optimized dilutions for protein detection. A mouse monoclonal antibody was used to detect beta-actin (Cell Signaling). Proteins were visualized using enhanced chemiluminescence (ECL) detection agents (Perkin Elmer).

### mRNA expression

Xenografted tumors were lysed using a Precellys Evolution Tissue Homogenizer with Cryolys Cooling Reservoir. mRNA was extracted using the Qiagen RNeasy Mini Kit (Qiagen), and mRNA was quantified with a NanoDrop 2000 instrument. Bio-Rad’s iScript cDNA Synthesis Kit (Bio-Rad) was used to generate a cDNA library and iTaq™ Universal SYBR^**®**^ Green Supermix was used for quantitative detection of transcripts via RT-PCR. Gene expression was evaluated by ΔΔct and normalized to B2M.

### shRNA knockdown

293T packaging cells were seeded at 1.0 × 10^6^ in 60 mm culture dishes and incubated for 24 hours. PSPAX2 lentiviral packaging plasmid (#12260), pMD2.g vsv-g envelope plasmid (#12259), and scramble shRNA control (#1864) were purchased from Addgene. HDAC2 shRNA were purchased from Sigma (SHCLNG-NM_001527). 2.5 ug PSPAX2, 2.5 ug pMD2.G, 5 ug shRNA, 250 uL serum free DMEM, and 20 uL Fugene 6 (Promega E2691) were added to a 1.7 mL tube and set for 30 minutes at room temperature. The reaction mixture was added to the 60mm dishes and incubated at 37 degrees for 18 hours. Media was replaced with DMEM containing 30% FBS and incubated for another 72 hours. The supernatant was collected and filtered through a 0.45 uM cellulose acetate membrane. To a 100 mm dish, LPS246 were seeded at 1.0 × 10^6^. Thirty minutes prior to infection, polybrene was added to the plate for a final concentration of 8 ug/mL. 1mL of the filtered viral supernatant was added to each plate according to each intended treatment. After 72 hours, the media was replaced and puromycin was added to reach a final concentration of 3.0 ug/mL. Following another 72 hours, the cells were collected for analysis.

### DDLPS xenograft model

Xenograft models were generated utilizing bilateral flank injections of the LPS863 cell line in 8-week-old, outbred, athymic nude (NCr-nu/nu) female mice. These mice were acquired from the athymic nude mouse colony maintained by the Target Validation Shared Resource (TVSR) at the Ohio State University; original breeders (strain #553 and 554) for the colony were received from the NCI Frederic facility. Bilateral flanks were injected with 1.5 × 10^6^ cells mixed in PBS:Matrigel (2:1, v/v) with a final volume of 150 μL. Once palpable tumors were established, mice were randomly divided into two treatment arms: vehicle control or romidepsin treated. Romidepsin (2 mg/kg) was administered twice weekly by intraperitoneal (IP) injection. Tumor and body weight measurements were performed every 3–4 days until animals were harvested on day 22 as planned prior to study initiation. At harvest, tumors were weighed, measured and prepared for mRNA expression analysis as described. All animal experiments were carried out under protocols approved by the Ohio State University Institutional Animal Care and Use.

### RNA sequencing

Four tumors from LPS863 xenografted athymic nude (NCr-nu/nu) female mice were randomly selected at the time of tumor harvest for both control and romidepsin treated mice as described above. Tumors were lysed using a Precellys Evolution Tissue Homogenizer with Cryolys Cooling Reservoir. mRNA was extracted using the Qiagen RNeasy Mini Kit (Qiagen) and treat with DNase I (Thermofisher). mRNA was quantified with a NanoDrop 2000 instrument. Using 200 ug RNA, a cDNA library was generated with NEBNext Ultra II Directional RNA Library Prep Kit for Illumina. An Illumina Hiseq 4000 was used to generate 17–20 × 10^6^ reads that passed filter cluster. Paired ends were 150 base pairs in length.

### Statistical analysis

All data was analyzed in Graphpad Prism v.8.0.0. Spearman’s correlation was used to assess co-expression between genes. Survival curves were calculated using the log-rank test for significance and cox proportional hazard regression to calculate hazard ratios (HR) with 95% confidence intervals (95%CI). Assessment of *HDAC2* as a continuous predictor of clinical outcomes was assessed individually for both datasets. In addition, two groups of patients with high (*HDAC2* High) and low (*HDAC2* Low) *HDAC2* expression were delineated using maximally selected rank statistics as implemented in the maxstat R package (http://cran.r-project.org/web/packages/maxstat/index.html). Student’s *t*-test was used as appropriate. The IC_50_ dose response curves were calculated in utilizing a four-parameter, Hill-slope equation. Ingenuity Pathway Analysis (IPA, QIAGEN) was used to conduct upstream regulator analysis. All data are reported as mean ± SEM unless otherwise noted; *p* values <0.05 were considered significant. All *in vitro* data was replicated in ≥2 independent experiments.

## SUPPLEMENTARY MATERIALS


