# Supplementary material for: Inhibition of histone deacetylase 2 reduces MDM2 expression and reduces tumor growth in dedifferentiated liposarcoma
Source: Oncotarget. 2019 Oct 1;10(55):5671–9. doi: 10.18632/oncotarget.27144 (PMC6779286; doi:10.18632/oncotarget.27144)
Supplement: Supplementary file 1 [file oncotarget-10-5671-s001.pdf]

Inhibition of histone deacetylase 2 reduces MDM2 expression and reduces tumor growth in dedifferentiated liposarcoma

SUPPLEMENTARY MATERIALS

Supplementary Table 1: Upstream regulator analysis results

Gene	Expression *p*-value	*p*-value of Overlap
IFNL1	-	2.19×10^{-19}
TGFB1	0.54	4.43×10^{-17}
TP53	0.001	4.75×10^{-17}
PRL	-	5.00×10^{-15}
ESR1	0.25	9.06×10^{-15}

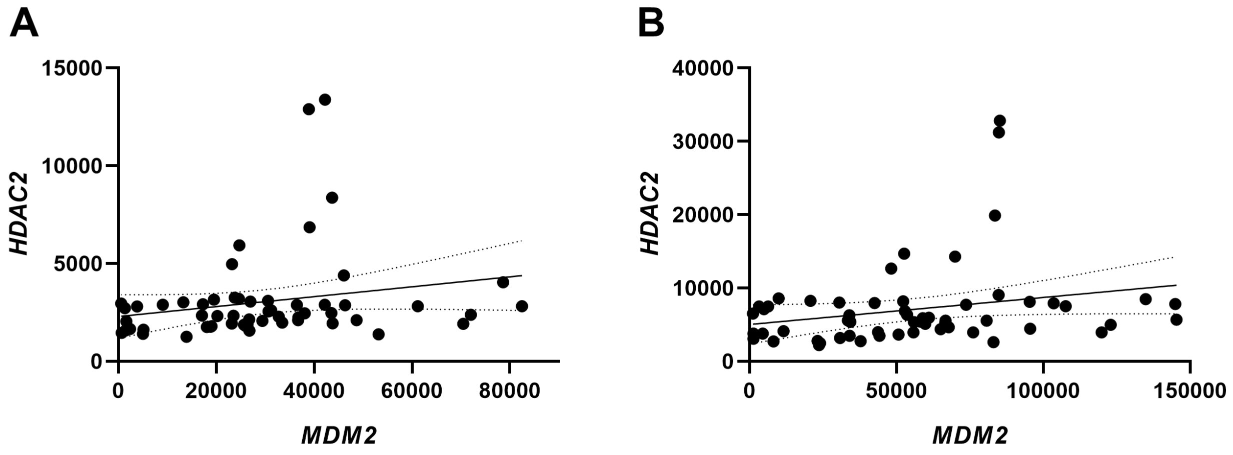

Supplementary Figure 1:Co-expression of *HDAC2* and *MDM2*. In both the TCGA (**A**) and MSKCC (**B**) datasets, expression of *HDAC2* was most highly correlated HDAC gene co-expressed with *MDM2*. In both datasets, *HDAC2* expression was positively correlated with *MDM2* expression (TCGA: Spearman's coefficient = 0.29, $p = 0.03$; MSKCC: Spearman's coefficient = 0.57, $p < 0.001$).

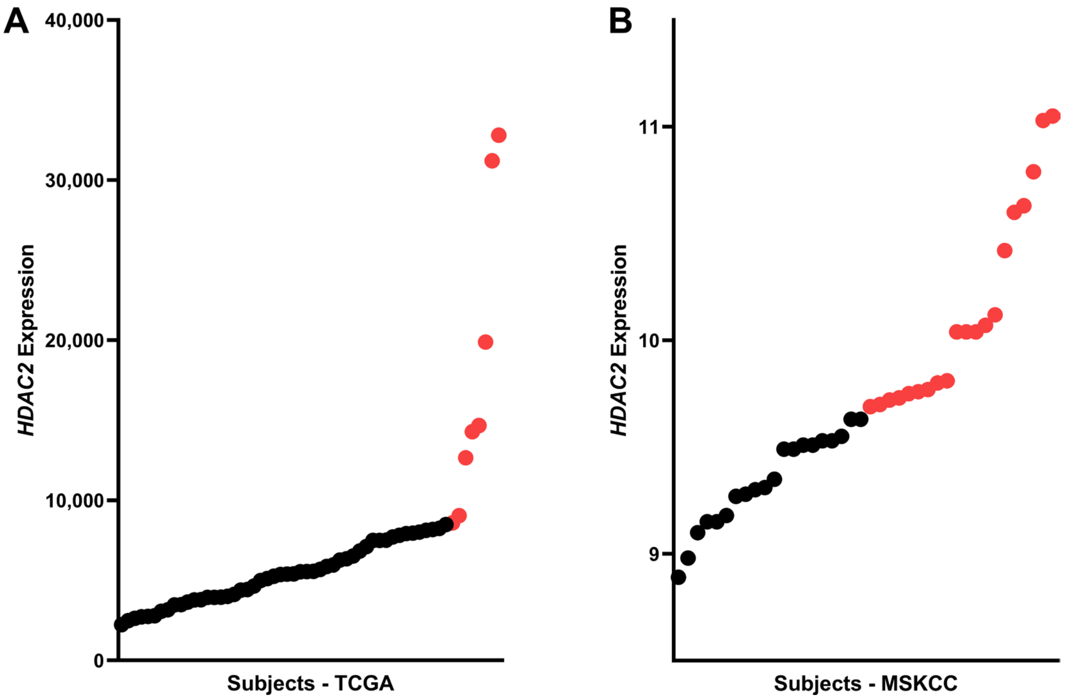

Supplementary Figure 2: *HDAC2* mRNA expression in clinical DDLPS samples. To assess the prediction of DFS OS by *HDAC2* mRNA expression as a dichotomous variable, subjects were split into *HDAC2* High (red points) and *HDAC2* Low (black points) groups utilizing maximally selected rank statistics for each dataset. mRNA expression of *HDAC2* as measured in the Cancer Genome Atlas (TCGA) by RNA-Seq (**A**). mRNA expression of *HDAC2* as measured in the Memorial Sloan-Kettering Cancer Center Dataset (MSKCC) by microarray (**B**).

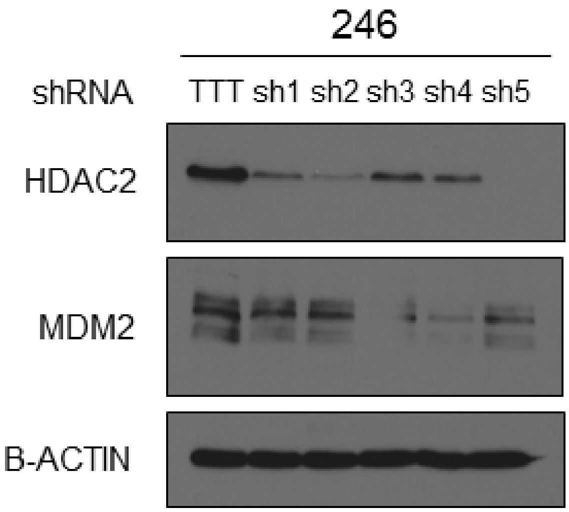

Supplementary Figure 3: shRNA knockdown of *HDAC2*. The LPS246 DDLPS cell line was incubated with lentiviral based scrambled *shRNA* (TTT) or *shRNA* MDM2-targeted vector (repeated five times, sh1-5). Cells underwent puromycin selection for 72 hours followed by 72 hours of incubation in normal media. Cells were then isolated for western blot analysis. Knockdown of *HDAC2* was successful and resulted in a reduction of MDM2 and p21 expression.
